# Assessment of the Thyroid Profile in the Iberian Lynx (*Lynx pardinus*)

**DOI:** 10.3390/vetsci13030278

**Published:** 2026-03-17

**Authors:** Adriana Maia, Rodrigo Serra, Ana C. Silvestre-Ferreira, Jaume Ródon, Guillermo López, Felisbina Pereira Queiroga

**Affiliations:** 1 Departamento de Ciências Veterinárias, Universidade de Trás-os-Montes e Alto Douro, Quinta de Prados, 5000-801 Vila Real, Portugal; admaia@hotmail.com; 2 Centro Nacional de Reprodução de Lince Ibérico, Estrada da Barragem do Funcho Km 4, 8375-082 São Bartolomeu de Messines, Portugal; rserra@cnrli.independentwildlife.eu; 3 Centro de Ciência Animal and Veterinary (CECAV), University of Trás-os-Montes e Alto Douro, Quinta dos Prados, 5000-801 Vila Real, Portugal; 4 Idexx Laboratories, Carrer del Plom, 2-8, 3rd Floor, 08038 Barcelona, Spain; jaume-rodon@idexx.com; 5 Agencia de Medio Ambiente y Agua de Andalucía, Avda. Lope de Vega 9, 29010 Málaga, Spain; guillermo.lopez.zamora@juntadeandalucia.es

**Keywords:** conservation, *Lynx pardinus*, Thyroid, hormones, reference interval, sex, age, environmental status

## Abstract

The Iberian lynx (*Lynx pardinus*), once considered the most endangered felid, has shown demographic recovery thanks to intensive conservation measures. Over the past two decades, large physiological datasets have supported both clinical management and research, yet thyroid function has remained comparatively underexplored. This study provides the first reference intervals (RI) for total thyroxine (TT4) and thyroid-stimulating hormone (TSH) in captive and wild lynxes, and assesses the effects of age, sex, and environment. Serum samples from 71 individuals (32 captive, 39 wild; 32 females, 39 males) were analysed, including 32 young adults, 14 adults, and 25 geriatric animals. TT4 was measured by enzyme immunoassay, and TSH using chemiluminescence. TT4 RI was 0.80–2.00 µg/dl (captive) and 0.70–2.20 µg/dl (wild) (*p* = 0.065). TSH RI was 0.00–1.10 ng/ml (captive) and 0.00–0.10 ng/ml (wild), showing a significant difference (*p* < 0.001). Captive males had higher TT4 than females (*p* = 0.018), while no sex difference appeared in wild lynxes (*p* = 0.408). Age had no significant effect on TT4 (*p* = 0.462) or TSH (*p* = 0.739). Findings confirmed that environment and sex (captivity) influenced thyroid parameters, while age did not. These values are crucial for health monitoring and endocrine assessment in this endangered species.

## Simple Summary

The Iberian lynx (*Lynx pardinus*), once considered the world’s most endangered wild cat, has made an impressive recovery thanks to strong conservation efforts. Over the past two decades, researchers have collected extensive health data to guide management and protect the species. However, until now, thyroid function had never been studied. This research provides the first reference values for thyroid hormones in Iberian lynx, comparing animals living in captivity with those in the wild. Blood samples were collected from lynxes of different ages and both sexes. The study found that the environment (captive or wild) and sex, in the case of captive animals, could influence thyroid activity. Age, on the other hand, did not appear to play a role. By establishing these baseline values, clinicians now have an important tool to monitor the health of the Iberian lynx more effectively. Understanding thyroid function adds a new dimension to conservation work, helping ensure that this iconic species continues its path toward survival and long-term stability.

## 1. Introduction

The Iberian lynx (*Lynx pardinus*) is an Iberian endemic felid restricted to the Iberian Peninsula [1]. Human persecution led to a rapid decline throughout the 20th century, and by 2002, only 94 individuals remained [2]. A comprehensive conservation program was then implemented, bringing together efforts from Portugal and Spain and integrating both in situ and ex situ measures, which ultimately reversed the previous negative trend [3,4]. The in situ measures were targeted at areas where wild Iberian lynx populations were known to exist or had previously existed, as well as at areas with potential for the species to survive and thrive. The Iberian Lynx Ex Situ Conservation Program took place in five Captive Breeding Centres of the Iberian Peninsula, playing a central role not only in population reinforcement but also in health monitoring, research, and management. As of 2022, the Iberian lynx population exceeded 2500 individuals [5], and the species was downlisted to Vulnerable in the IUCN “Red List” (International Union for Conservation of Nature’s Red List of Threatened Species) [6].

Despite this remarkable recovery, Iberian lynx conservation continued to represent a major ecological and veterinary challenge. The species remained vulnerable due to the complex interplay between ecological fragility, reduced genetic diversity, disease susceptibility, and ongoing anthropogenic pressures [7]. Many of these pressures persisted or even intensified due to human activities, both directly and indirectly, pushing the species into an extinction vortex that proved difficult to reverse [8]. In this context, health surveillance has become an increasingly important component of conservation strategies, as subclinical disease and physiological imbalance can compromise individual fitness and long-term population viability.

At the beginning of the 21st century, basic clinical information about the Iberian lynx was scarce or non-existent. Since then, the Iberian lynx has been extensively studied, and establishing biomedical databases and baseline reference values for clinical parameters of wild and captive lynxes has been a high priority for both Iberian governments (Spanish and Portuguese). In the early 1990s, haematological and biochemical parameters were characterized in a population of *L. pardinus* in Spain [9]. By 2010, serum biochemical reference intervals had been established for a broader sample of the species [10]. More recently, in 2021, another study identified the blood groups of the Iberian lynx for the first time [11]. Since its inception, the ex situ conservation program has devoted substantial effort to these determinations [4], contributing significantly to the standardization of clinical assessment and health monitoring in the species. One of the consequences of the successful conservation strategy was a growing population that included a larger number of aging individuals, a scenario rare in the wild and more evident in captivity. This demographic shift brought new clinical concerns, such as the need to understand age-related endocrine changes, particularly those involving thyroid hormones, which are essential regulators of metabolism, thermoregulation, and behaviour [4]. In domestic cats, advancing age has been frequently associated with an increased risk of hyperthyroidism, which can manifest with behavioural signs such as restlessness, aggression, and hyperactivity, as well as clinical signs including weight loss despite normal or increased appetite, and cardiovascular abnormalities [12]. In the context of wildlife conservation, these subtle or progressive clinical signs can easily go unnoticed or be misinterpreted as stress-related behaviors or environmental adaptation. This diagnostic uncertainty underscores the importance of objective laboratory parameters to support clinical decision-making in both captive and free-ranging individuals. Thyroid neoplastic lesions have been reported in a leopard (*Panthera pardus*) [13] and in other wild felids [14,15]; however, a clear association with overt thyroid-related clinical signs has not been supported by the available evidence.

Without established reference intervals for thyroid hormones in *L. pardinus*, it is not possible to reliably identify abnormal or pathological conditions, nor to distinguish them from physiological variation. The absence of such data represents a limiting factor in both clinical and conservation medicine. Consequently, the main objective of this study is to establish the first species-specific reference intervals (RI) for total thyroxine (TT4) and thyroid-stimulating hormone (TSH) in the Iberian lynx, and thus to investigate the potential influence of environmental status (captive vs. wild), sex, and age on these hormonal parameters. By addressing this knowledge gap, the present study aims to provide a robust framework for endocrine evaluation, offering a tool readily applicable to routine health surveillance and long-term management programs. This information provides valuable support for long-term clinical surveillance and welfare in this critically endangered yet important species.

## 2. Materials and Methods

### 2.1. Animals

Blood samples were collected from both wild and captive clinically healthy Iberian lynxes (*L. pardinus*). Captive individuals included in this study were subjected to daily clinical monitoring by trained staff at each Breeding Centre, allowing early recognition of subtle behavioral or physiological changes. In contrast, health assessment of wild individuals was inherently more challenging, relying primarily on indirect information obtained through camera trapping and telemetry, with clinical evaluation only becoming possible when animals were physically captured and sedated [16].

Given the species’ strict ecological requirements, particular emphasis was placed on replicating natural conditions as closely as possible in captivity, especially regarding diet. The Iberian lynx is a highly specialized predator, with the European rabbit (*Oryctolagus cuniculus*) accounting for approximately 80–90% of its natural diet. Captive animals were fed geese (*Anser* spp.), ducks (*Anas platyrhynchos*), and hares (*Lepus europaeus*), according to structured feeding protocols. Breeding centres implemented feeding schedules aligned with the species’ circadian rhythm and prey preferences, ensuring controlled rotation and dietary diversity [17].

The wild animals were from both Sierra Morena’s and Doñana’s populations, and their selection was as random as the animals captured using live traps. The captive group belonged to the Spanish Breeding Centres of Granadilla, Acebuche, and La Olivilla. The included animals were of both sexes and ranged from 3 to 11 years of age. Age classification was established using expert knowledge of Iberian lynx biology and husbandry and aligned with the guidelines of the Iberian Lynx Captive Management Manual [17]. In this species, individuals are generally considered adults from approximately 3 years of age onwards, with adulthood primarily defined by sexual and behavioural maturity rather than body size alone. For this study, and to explore potential clinically relevant differences within the adult population, individuals aged 3–8 years were classified as adults and further subdivided into young adults (3–5 years) and mature adults (6–8 years). Animals aged 9 years or older were classified as geriatric, as they had surpassed approximately 70–80% of the species’ expected lifespan and might exhibit age-related physiological decline. The average life expectancy of the Iberian lynx in the wild is approximately 13 years, with captive individuals occasionally reaching, but rarely exceeding, this age [17].

The study included 71 Iberian lynxes, of which 32 were captive and 39 were wild. The sample consisted of 32 females and 39 males, with ages spanning 3 to 11 years. The distribution of age groups and sex across population settings is summarized in Table 1.

### 2.2. Ethical Approval

This study was conducted under a protocol established between the Universidade de Trás-os-Montes e Alto Douro (UTAD) and the Portuguese Breeding Centre of Iberian lynx (CNRLI) (Protocol nº 1319-e-CITAB-2021). The collection and use of biological samples (serum) from *L. pardinus* were conducted in accordance with Spanish and Andalusian regulations on wildlife protection and biodiversity conservation (Article 9 of Law 8/2003, of 28 October, on Wild Flora and Fauna). The study was approved and authorized by the Consejería de Agricultura, Ganadería, Pesca y Desarrollo Sostenible, Junta de Andalucía, under the Resolution issued on 10 February 2022. This authorization covered the use of serum samples from healthy captive and wild Iberian lynx (*L. pardinus*).

### 2.3. Samples

Blood samples were collected between July 2015 and December 2021 during routine health check-ups. Check-ups followed clinical procedures specifically developed for the species as reflected in the Iberian lynx Health Management Manual v2.1 [16]. All animals in this study were physically restrained prior to the anaesthesia using capture cages. According to the mentioned guidelines, all anesthetic procedures, excluding emergencies, were conducted in clinics incorporated in each specialized Breeding Centre, to mitigate avoidable complications. Consequently, each animal was physically restrained in a transport cage during the transfer to the nearest Specialized Centre, ensuring the lowest possible stress levels. As all animals included in the study were previously deemed clinically healthy upon capture, the anaesthetic protocol followed the recommendations for healthy animals outlined in the Iberian lynx Health Management Manual v2.1 [16]: a combination of alpha-2 adrenergic receptors agonist (commonly dexmedetomidine at 0.025 mg/kg) and ketamine (5 mg/kg), to which other drugs can be added as needed (midazolam, diazepam, methadone, morphine, fentanyl or buprenorphine, according to each situation) [16]. At this point, the animals underwent a complete clinical examination, confirming their overall health status. According to the same manual, blood samples were collected from the cephalic, saphenous, or jugular veins using 5–10 mL syringes, 21G–19G butterfly needles, and serum separation tubes with clot activator (FL Medical, Torreglia, Italy). The blood samples underwent standard centrifugation at 800× *g* for 5 min to separate the serum [16]. Serum was transferred into labelled aliquots and stored at −80 °C, remaining at the Junta de Andalucía facilities in Spain until analysis in April 2022. The samples were transported to the laboratory under controlled time and temperature conditions to ensure their preservation.

### 2.4. Hormonal Determination

Total T4 (TT4) was measured using an enzyme immunoassay (DRI Thyroxine Assay, Microgenics Corporation, Fremont, CA, USA) on a Beckman Coulter AU5800 Series chemistry analyzer. The analytical measurement range for TT4 was 0.5–20 µg/dL, and the assay was linear from 0.44 to 11.73 µg/dL (5.66–150.99 nmol/L), with reported intra- and inter-assay coefficients of variation <12%.

Serum TSH concentrations were measured using a chemiluminescent immunoassay (IMMULITE Canine TSH, Siemens Healthcare Diagnostics, Tarrytown, NY, USA) on the IMMULITE 2000 XPi Immunoassay System, with an analytical measurement range of 0.03–12 ng/mL and a sensitivity of 0.01 ng/mL. Intra- and inter-assay coefficients of variation for TSH were concentration dependent. All hormonal analyses were conducted at IDEXX Laboratories (Barcelona, Spain). Both assays have been previously validated and were widely used in veterinary diagnostics [18].

### 2.5. Statistical Analysis

Descriptive analyses and reference interval (RI) estimation for TT4 and TSH were conducted using Reference Value Advisor v2.1, a Microsoft Excel-based macro for RI computation [19]. This software applied the Anderson–Darling test to assess data normality and identified outliers using the Tukey and Dixon–Reed methods. According to the data distribution, parametric or robust methods, with or without Box–Cox transformation, were applied. Reference intervals and their 90% confidence intervals (CI) for the lower and upper limits were subsequently calculated. RI estimation strictly followed the American Society of Veterinary Clinical Pathology (ASVCP) guidelines [20], taking into account the study’s sample size. As parametric methods estimate population-based limits, calculated RI could extend beyond the observed data range; lower limits were truncated at zero when required for physiological plausibility.

Analyses assessing potential differences by sex and age group were conducted using SPSS software (version 30.0). Data normality was evaluated with the Shapiro–Wilk and Kolmogorov–Smirnov tests. Depending on distribution, comparisons were performed using one-way ANOVA or non-parametric tests (Kruskal–Wallis or Mann–Whitney U). Statistical significance was set at *p* < 0.05.

## 3. Results

For total thyroxine (TT4), the RI was calculated using all 39 wild individuals and 31 captive individuals, as one captive sample had insufficient volume. Results are shown in Table 2. For thyroid-stimulating hormone (TSH), the RI was calculated from 38 wild and 29 captive animals due to insufficient sample volume (Table 2). According to ASVCP guidelines [20], the sample sizes used (31 captive and 39 wild lynx) fall below the recommended threshold for standard reference interval estimation. Therefore, the resulting reference intervals must be interpreted with caution, and the 90% confidence interval limitations were explicitly reported to reflect the increased uncertainty associated with the relatively small sample sizes.

### 3.1. Effect of Sex and Age on TT4 and TSH

In both captive and wild populations, TT4 concentrations were significantly influenced by sex (*p* = 0.024 and *p* = 0.023, respectively), with males presenting higher mean values than females (Table 3). In contrast, the TSH levels were not significantly affected by sex in either population (*p* = 0.808 in captive animals and *p* = 0.395 in wild animals).

Age did not appear to significantly influence the concentrations of either TT4 or TSHs in the studied captive population. Although the mean values for both hormones were higher in the geriatric group compared to the younger groups, the differences were not statistically significant (*p* = 0.066 for TSH and *p* = 0.054 for TT4). Similarly to the findings in the captive population, age did not appear to significantly influence the concentrations of either hormone in the wild group. Although mean TT4 values varied across age groups, the differences were not statistically significant (*p* = 0.095 for TT4; *p* = 0.630 for TSH), (Table 4).

### 3.2. Comparison Between Captive and Wild Populations

For both TT4 and TSH, wild animals showed significantly lower mean values than captive individuals (*p* < 0.01) (Table 5). For TT4, one sample had insufficient volume for analysis, and for TSH, four samples also had insufficient volume.

## 4. Discussion

Since 2015, the Iberian lynx (*L. pardinus*) has been considered an endangered species according to IUCN criteria [21]. Even though there has been an improvement from the previous classification, it still reflects the small number of animals of the species in both Portugal and Spain. The conservation and recovery program for the Iberian lynx is relatively recent, which explains the lack of information in several clinical areas, including the thyroid profile. Moreover, conservation efforts have resulted in an increasingly older Iberian lynx population, a factor known to influence thyroid function and the prevalence of thyroid diseases in other species [12,22]. In domestic cats, aging has been associated not only with an increased prevalence of thyroid disease, but also with subtle shifts in thyroid hormone concentrations that may precede overt clinical dysfunction, highlighting the relevance of endocrine monitoring in aging populations [12,22].

To the best of the authors’ knowledge, thyroid function has not previously been studied in the Iberian lynx, and this research provides the first RI for TT4 and TSH in this species, providing a necessary baseline to distinguish physiological variation from pathological alterations in both free-ranging and managed individuals.

The differences in mean TT4 and TSH values between captive and wild animals were statistically significant, with captive individuals showing notably higher levels. The TT4 results obtained in this study were consistent with current literature for domestic cats, showing a higher prevalence of elevated thyroid hormone values in indoor animals compared to outdoor cats [23]. It has been suggested that construction materials containing certain chemical compounds, such as polybrominated diphenyl ethers (PBDEs), may stimulate the thyroid gland in domestic cats [22,24,25,26], potentially explaining the increased TT4 levels observed in our captive animals. Some authors have demonstrated a positive correlation between PBDE levels in house dust and serum thyroid concentrations in hyperthyroidic domestic cats [27,28]. This hypothesis may also apply to the captive Iberian lynx included in this study; further investigation is needed to support this assumption, including the assessment of PBDE levels in enclosure environments and their comparison with serum concentrations. Additionally, some studies have shown a relationship between thyroid dysfunction in cats and exposure to pesticides, herbicides, and fertilizers [23]. However, this potential risk was not evaluated in the present study and therefore cannot be considered.

Another nutritional factor that may influence thyroid hormone concentrations is iodine intake, as iodine is an essential component of thyroid hormone synthesis. Although captive Iberian lynxes were fed prey-based diets designed to replicate their natural feeding ecology, the iodine content of captive diets and wild prey was not quantified in the present study. Consequently, potential differences in iodine intake between captive and wild animals cannot be completely excluded. However, previous studies in domestic cats suggest that thyroid hormone variability is usually influenced by multiple environmental and physiological factors rather than by a single determinant [22,23]. Future studies incorporating direct assessment of dietary iodine levels in captive feed and natural prey would help clarify the possible contribution of nutritional factors to thyroid function in this species.

Regarding TSH, the available literature in domestic cats remains limited, making it difficult to directly compare the findings of the present study in wild and captive Iberian lynx with those reported for other felid species. Emotion-related factors such as anxiety and excitement have been reported to reduce TSH secretion in humans [29]. Although this association has been primarily investigated in humans, it remains relevant to the present study, as stress is commonly linked to captivity. These factors could theoretically be more prevalent in captive animals due to confinement or excitement related to interactions with keepers. Interestingly, the present study reported the opposite pattern, with higher TSH concentrations observed in captive animals. Diet has also been implicated in variations in thyroid hormone levels in domestic cats, mainly in relation to fluctuations in iodine intake [22,29].

Nonetheless, the strict and varied dietary regimen implemented in the captive Breeding Centres for *L. pardinus* [17] makes diet an unlikely sole explanation for the higher TT4 levels observed in this group. This supports the hypothesis that thyroid function is shaped by the synergistic action of multiple environmental and management-related factors, rather than by any single determinant [22]. Additional hypotheses have been proposed to explain the higher thyroid hormone levels observed in indoor domestic cats, including the use of litter trays. None of these theories has yet been proven in cats, and they do not apply to the present study, as the captive populations are managed within rigorous breeding programs at specialized centers that aim to replicate the species’ natural ecology and do not employ the materials implicated in these hypotheses [27].

In domestic cats, an increased risk of hyperthyroidism has been reported in females [30,31]; however, other epidemiological studies have not identified this association as statistically significant [32,33]. Moreover, additional studies on domestic cats have documented higher TT4 concentrations in males compared to females [34,35]. A study in wild felids determined T3 and T4 concentrations in nine species and evaluated the influence of sex on these hormones in four of those species, with no significant differences found [36]. Similarly, another study established reference thyroid hormone values for Cheetahs and observed no significant differences between sexes [37]. The heterogeneity of these results across felid species suggests that sex-related modulation of thyroid function may be species-specific and influenced by environmental context, rather than representing a conserved biological pattern.

Nevertheless, the controversial published studies on thyroid function according to sex in felids in general led the authors to consider insights from previous studies performed with humans, suggesting an association between thyroid function and the female reproductive system, indicating that estrogens could increase TSH levels and subsequently TT4 [38]. Our study’s findings in *L. pardinus* align with some of the studies reported for domestic cats, demonstrating higher TT4 levels in males and no significant influence of sex on TSH concentrations. The relationship between thyroid hormones and sexual behavior could not be assessed in the present study, as neither behavioral parameters nor reproductive hormones were evaluated in Iberian lynx. In addition, although the present study evaluated the effects of sex and age within each environmental group, potential interaction effects between environmental status and these biological variables could not be robustly assessed due to the limited sample size and the uneven distribution of individuals among subgroups. Future studies with larger and more balanced datasets would allow the application of multivariable models to better explore these potential interactions.

Regarding age, no statistically significant differences were observed in either population. However, certain trends were noted, such as slightly lower TT4 levels in geriatric wild animals, although these differences were not statistically significant. These results align with previous findings that reported decreased thyroid hormone concentrations in older cats [34]. Given that all animals included in this study were considered clinically healthy, these findings likely reflect physiological aging rather than pathological thyroid dysfunction. Nevertheless, it is essential to emphasize that all animals included in the current study were previously considered clinically healthy, precluding direct extrapolations regarding age-related pathological conditions.

One limitation of this study is the absence of detailed information regarding the exact time of day and season when samples were collected. Thyroid hormone concentrations may vary according to physiological factors such as circadian rhythms, seasonal influences, and reproductive status in mammals, including felids [12,29]. Because samples in the present study were collected opportunistically during routine clinical procedures and health monitoring of the animals, these temporal variables could not be standardized or evaluated. Consequently, their potential influence on hormone concentrations cannot be completely excluded and should be considered when interpreting the results. Another limitation of this study is the uneven distribution of samples across age groups within both wild and captive populations. Future studies employing more homogeneous sample distributions may yield more definitive conclusions on the influence of age on thyroid function in *L. pardinus*.

## 5. Conclusions

This study establishes the first reference intervals for TT4 and TSH in *L. pardinus*, providing essential baseline data for evaluating thyroid function in this endangered species. Environmental status significantly influenced TSH and TT4 levels, with captive animals showing notably higher values. Sex also had a significant effect on TT4, as males exhibited higher concentrations than females, whereas no sex-related differences were detected for TSH. Age did not significantly affect the levels of either hormone. The relationship between age and thyroid function in Iberian lynx was consistent with the existing literature for domestic cats. To some extent, the patterns observed for sex and thyroid hormone concentrations were consistent with what has been reported in domestic cats, but they did not align with the limited literature available for wild felids. The environmental status showed a clear parallel with findings previously reported for domestic cats (indoors vs. outdoors); however, it also raised additional questions that warrant further investigation.

The findings of this study advance the understanding of thyroid function in the endangered *L. pardinus* and provide a valuable foundation for future health monitoring and conservation strategies.

## Figures and Tables

**Table 1:** Demographic characteristics of captive and wild Iberian lynx (*Lynx pardinus*) included in the study.

Variable	Captive (n = 32)	Wild (n = 39)	Total (n = 71)
**Sex**			
**Females, n (%)**	16 (50%)	16 (41%)	32 (45%)
**Males, n (%)**	16 (50%)	23 (59%)	39 (55%)
**Age**			
**Young adults (3–5 years), n (%)**	9 (28%)	23 (59%)	32 (45%)
**Adults (6–8 years), n (%)**	8 (25%)	6 (15%)	14 (20%)
**Geriatrics (9–11 years), n (%)**	15 (47%)	10 (26%)	25 (35%)

Age groups were defined as follows: young adults (3–5 years), adults (6–8 years), and geriatric (≥9 years). Percentages are calculated within each population.

**Table 2:** Reference intervals for total thyroxine (TT4) and thyroid-stimulating hormone (TSH) in healthy captive and wild Iberian lynx (*Lynx pardinus*).

	Parameter	n	Mean	SD	Median	Min–Max	RI	LRL 90% CI	URL 90% CI
**Captive**	**TT4 (µg/dL)**	31	1.37	0.30	1.30	0.70–2.00	0.80–2.00	0.70–0.90	1.80–2.20
	**TSH (ng/mL)**	29	0.22	0.21	0.14	0.03–0.80	0.00–1.10	0.00–0.00	0.60–1.80
**Wild**	**TT4 (µg/dL)**	39	1.22	0.35	1.10	0.70–2.40	0.70–2.20	0.60–0.80	1.90–2.60
	**TSH (ng/mL)**	38	0.04	0.02	0.03	0.03–0.14	0.00–0.10	0.00–0.00	0.00–0.10

n = sample size; SD—standard deviation; Min—Minimum value detected; Max—Maximum value detected; RI—reference interval; LRL 90% CI—Lower Real Limit at a Confidence Interval of 90%; URL 90% CI—Upper Real Limit at a Confidence Interval of 90%.

**Table 3:** Total thyroxine (TT4) and thyroid-stimulating hormone (TSH) concentrations by sex in healthy captive and wild Iberian lynx (*Lynx pardinus*).

Population	Hormone	Sex	n	Mean ± SD * or Median **	Min–Max	*p*-Value
**Captive**	**TT4 (µg/dL)**	Female	16	1.26 ± 0.24	0.70–1.60	0.024
		Male	15	1.49 ± 0.30	1.10–2.00	
	**TSH (ng/mL)**	Female	15	0.15	0.03–0.8	0.896
		Male	14	0.13	0.03–0.55	
**Wild**	**TT4 (µg/dL)**	Female	16	1.00	0.70–1.40	0.033
		Male	23	1.30	0.80–2.40	
	**TSH (ng/mL)**	Female	15	0.03	0.03–0.05	0.847
		Male	23	0.03	0.03–0.14	

n = sample size; SD—standard deviation; Min—Minimum value detected; Max—Maximum value detected. * Mean values were used for parameters with normal distribution; ** Median values were used for parameters with non-parametric distribution.

**Table 4:** Age effect on total thyroxine (TT4) and thyroid-stimulating hormone (TSH) values in healthy captive and wild populations of Iberian lynx (*Lynx pardinus*).

Population	Hormone	Age Group	n	Mean ± SD * or Median **	Min–Max	*p*-Value
Captive	TT4 (µg/d L)	3–5 (young adults)	9	1.26 ± 0.19	0.90–1.60	
		6–8 (adults)	8	1.26 ± 0.36	0.70–1.90	0.066
		9–11 (geriatrics)	14	1.50 ± 0.26	1.10–2.00	
	TSH (ng/m L)	3–5 (young adults)	9	0.13	0.05–0.27	
		6–8 (adults)	8	0.13	0.03–0.46	0.284
		9–11 (geriatrics)	12	0.24	0.03–0.80	
Wild	TT4 (µg/d L)	3–5 (young adults)	23	1.10	0.70–1.90	
		6–8 (adults)	6	1.45	0.90–2.40	0.282
		9–11 (geriatrics)	10	1.10	0.80–1.60	
	TSH (ng/m L)	3–5 (young adults)	22	0.03	0.03–0.14	
		6–8 (adults)	6	0.03	0.03–0.05	0.762
		9–11 (geriatrics)	10	0.03	0.03–0.04	

n = sample size; SD—standard deviation; Min—Minimum value detected; Max—Maximum value detected. * Mean values were used for parameters with normal distribution; ** Median values were used for parameters with non-parametric distribution.

**Table 5:** Comparison of total thyroxine (TT4) and thyroid-stimulating hormone (TSH) concentrations between healthy captive and wild Iberian lynx (*Lynx pardinus*).

Hormone	Population Type	n	Median (Min–Max)	*p*-Value
TT4 (µg/dL)	Captive	31	1.30 (0.70–2.00)	0.023
	Wild	39	1.10 (0.70–2.40)	
TSH (ng/mL)	Captive	29	0.14 (0.03–0.80)	<0.001
	Wild	38	0.03 (0.03–0.14)	

n = sample size; Min—Minimum value detected; Max—Maximum value detected.

## Data Availability

The original contributions presented in this study are included in the article. Further inquiries can be directed to the corresponding authors.

## Abbreviations

The following abbreviations are used in this manuscript:

TT4	total thyroxine
TSH	thyroid-stimulating hormone
RI	reference interval
